# High Throughput Profiling of Flavonoid Abundance in *Agave lechuguilla* Residue-Valorizing under Explored Mexican Plant

**DOI:** 10.3390/plants10040695

**Published:** 2021-04-03

**Authors:** Zoé P. Morreeuw, David Castillo-Quiroz, Leopoldo J. Ríos-González, Raúl Martínez-Rincón, Norma Estrada, Elda M. Melchor-Martínez, Hafiz M. N. Iqbal, Roberto Parra-Saldívar, Ana G. Reyes

**Affiliations:** 1 Centro de Investigaciones Biológicas del Noroeste (CIBNOR), Instituto Politécnico Nacional 195, Playa Palo Santa Rita Sur, La Paz 23096, Mexico; zpelletier@pg.cibnor.mx; 2 Instituto Nacional de Investigaciones Forestales, Agrícolas y Pecuarias (INIFAP), Campo Experimental Saltillo, Carretera Saltillo-Zacatecas 9515, Col. Hacienda Buenavista, Saltillo 25315, Mexico; dacastilloq@gmail.com; 3 Departamento de Biotecnología, Facultad de Ciencias Químicas, Universidad Autónoma de Coahuila (UAdeC), Blvd. V. Carranza, Republica Oriente, Saltillo 25280, Mexico; leopoldo.rios@uadec.edu.mx; 4 Programa Catedra CONACYT-CIBNOR, Av. Instituto Politécnico Nacional 195, Playa Palo Santa Rita Sur, La Paz 23096, Mexico; rrincon@cibnor.mx (R.M.-R.); nestrada@cibnor.mx (N.E.); 5 Tecnologico de Monterrey, School of Engineering and Sciences, Monterrey 64849, Mexico; elda.melchor@tec.mx

**Keywords:** flavonoid concentrations, waste biomass, bioactive compounds, HPLC analysis, geographical variability, solvent extraction, storage conditions

## Abstract

*Agave lechuguilla* waste biomass (*guishe*) is an undervalued abundant plant material with natural active compounds such as flavonoids. Hence, the search and conservation of flavonoids through the different productive areas have to be studied to promote the use of this agro-residue for industrial purposes. In this work, we compared the proportion of total flavonoid content (TFC) among the total polyphenolics (TPC) and described the variation of specific flavonoid profiles (HPLC-UV-MS/MS) of *guishe* from three locations. Descriptive environmental analysis, using remote sensing, was used to understand the phytochemical variability among the productive regions. Furthermore, the effect of extractive solvent (ethanol and methanol) and storage conditions on specific flavonoid recovery were evaluated. The highest TPC (16.46 ± 1.09 GAE/g) was observed in the *guishe* from region 1, which also had a lower normalized difference water index (NDWI) and lower normalized difference vegetation index (NDVI). In contrast, the TFC was similar in the agro-residue from the three studied areas, suggesting that TFC is not affected by the studied environmental features. The highest TFC was found in the ethanolic extracts (6.32 ± 1.66 QE/g) compared to the methanolic extracts (3.81 ± 1.14 QE/g). Additionally, the highest diversity in flavonoids was found in the ethanolic extract of *guishe* from region 3, which presented an intermedia NDWI and a lower NDVI. Despite the geo-climatic induced variations of the phytochemical profiles, the results confirm that *guishe* is a valuable raw material in terms of its flavonoid-enriched bioactive extracts. Additionally, the bioactive flavonoids remain stable when the conditioned agro-residue was hermetically stored at room temperature in the dark for nine months. Finally, the results enabled the establishment of both agro-ecological and biotechnological implications.

## 1. Introduction

Inhabitants of arid and semi-arid regions of Mexico empirically harvest the *Agave lechuguilla* Torr. (Asparagaceae), locally known as lechuguilla, as a common-pool resource for its fiber (*ixtle*), which is used to manufacture diverse items, brushes, ropes, carpets, and textiles [1,2]. Since the 1970s, fiber exploitation has been intensified, mainly for exportation (93%), providing subsistence incomes to more than 20,000 farmer families [3,4,5]. However, the fiber is sold at low prices, which has added to the high marginalization degree and social backwardness, thus contributing to the poverty conditions in which these communities live [6,7]. *A. lechuguilla* is used in a large part of the Mexican territory, covering a diversity of geo-climatic conditions that impact growth and therefore the regional productivity [8,9]. For the conservation of *A. lechuguilla* wild populations, the Secretary of Environment and Natural Resources in Mexico established Official Standards, which only allows harvesting the central stem, known as *cogollo* [10]. Depending on the environmental conditions, between 12 and 24 months are needed for the *cogollo* to grow back up to 25 cm [9], which is the standardized size for harvest. As a result, the harvest of *A. lechuguilla* for fiber extraction has become a managed practice that can be considered sustainable [4,11]. The annual production of fiber is concentrated in six states of Mexico (San Luis Potosí, Coahuila, Nuevo León, Zacatecas, Durango, and Tamaulipas) and reaches around 55.98 kg/ha [12]. However, the manual or mechanical shredding of the leaves results in material consisting of 15% fiber (*ixtle*) and 85% wasted plant residue (*guishe*), representing over 150 thousand tons of residual biomass each year [2,13]. This abundant agro-residue does not have commercial value and is discarded, leading to ecological issues [2,9].

In this concern, there is a growing interest in the use of *A. lechuguilla* for the bioenergy industry [14,15,16,17]. The lechuguilla is also considered for uses in construction [18,19], soil and water decontamination [20,21], and bio-based polymers [22]. Likewise, other industrial fields, such as agricultural production [23,24,25,26], animal feed additives [27], agroindustry [28,29], nutraceuticals [30], and pharmacology [31,32,33,34,35] are interested in *A. lechuguilla*, especially for its active ingredients. Among the bioactive compounds of interest, Almaraz-Abarca et al. [36] reported the presence of phenolic acid derivatives, flavonols, dihydroflavonoids, and glycoside flavonoids in the leaf extracts of *A. lechuguilla*. Previous studies have demonstrated the antimicrobial and antifungal activity of polyphenols-rich extracts obtained from *A. lechuguilla* [24,25,28,37]. Similarly, the insecticidal capacity of the leaf extracts has been related to the presence of saponins, flavonoids, terpenes, and phenolic compounds [26]. In addition, the antioxidant capacity and anti-carcinogenic potential of the flavonoids found in *A. lechuguilla* ethanolic extracts have been evidenced [35]. However, most of these studies use the entire leaves of the *cogollo*, which is a disadvantage because of its competition with the fiber market [2,38]. Hence, the conceptualization of a biorefinery promotes the use of *A. lechuguilla* agro-residue to generate a wide range of high added-value co-products [2].

Focusing on the characterization of phytochemicals in *A. lechuguilla* agro-residue, our research group identified 53 compounds among the flavonols, anthocyanidins, flavanones, and isoflavonoids, with a significant abundance of their glycoside derivatives [39]. Moreover, the crude extract of the lechuguilla residue, tested as a bioactive ingredient for farmed shrimps, showed health benefits that were attributed to its saponin and flavonol content [27]. These previous results support the valorization proposal of this constantly produced agro-residue. However, future demand for *A. lechuguilla* should be associated with the implementation of commercial crops and biorefineries for its sustainable use. In this regard, our research group has successfully domesticated and cultivated this species of *Agave* on an experimental scale [9]. Furthermore, the effect of the geo-climatic factors and agronomic management on fiber productivity has already been demonstrated [5]. Additionally, the liquid fraction of the agro-residue from three localities showed different saponin concentrations, and this variation was attributed to divergent environmental stress [13]. In contrast, to our knowledge, the phenolic content of *A. lechuguilla* residue has not yet been investigated from a geographical perspective. The climatic features of arid and semi-arid areas are known for being a determinant in the accumulation patterns of specialized metabolites such as flavonoids [40]. However, the link between these environmental conditions and bioactive compounds in crassulacean acid metabolism (CAM) plants such as *A. lechuguilla* still has to be well established. Thus, understanding plant response to water availability, nutrients, and further abiotic and biotic conditions could provide valuable information for crop management. In addition to crop development within the biorefinery scheme [2], added-value products are needed to improve economic feasibility of the bioenergy processes. Therefore, the aim of this study was to compare the bioactive flavonoid contents of the lechuguilla agro-residue collected in three productive localities of northeastern Mexico. Total phenolic and flavonoid contents (TPC and TFC) analysis were complemented with a qualitative and quantitative HPLC-UV-MS/MS study to detect the potential bioactive flavonoids. The variability in flavonoid profiles was related to environmental features of the sampled regions, characterized through remote sensing. In addition, storage conditions and solvent extraction were studied as the first steps of agro-residue processing to meet industrial requirements.

## 2. Materials and Methods

### 2.1. Agro-Residue Collection

Plant material was obtained on August 2018 from: (1) Ejido Gómez Farías, Matehuala, San Luis Potosí, Mexico (GPS: 23°28′54.3″ N; 100°37′22.1″ W), (2) Ejido Cosme, Ramos Arizpe, Coahuila, Mexico (GPS: 25°52′03.6″ N; 101°19′51.1″ W), and (3) Ejido Tuxtepec, Ramos Arizpe, Coahuila, Mexico (GPS: 26°11′29.8″ N; 101°11′0.96″ W). Registered exploiters carried out the harvesting and processing of the central leaves of *Agave lechuguilla*, according to Mexico’s Official Standards for central stem harvesting and land shifts [10]. The agro-residue was collected during the mechanical fiber extraction and cryopreserved at −80 °C. Posteriorly, the *guishe* was freeze-dried for 48 h in the dark at −49 °C under vacuum (Labconco Corporation, Kansas City, MO, USA) and milled to a 2 mm particle size powder (Retsch-SM100 Industrial Mill, Haan, Germany). Moisture content was determined by weight reduction of 500 mg after 15 min exposure to 120 °C (Thermobalance MB45, OHAUS, Mexico).

### 2.2. Storage Conditions

The conditioned dry powder was stored at room temperature (<25 °C), in the absence of light, oxygen, and moisture, until phytochemical extractions were performing. A first characterization of the polyphenolic content was achieved after a negligible storage time (t0), whereas the rest of the agro-residue was preserved under the described storage conditions for 9 months (t9) before downstream analysis.

### 2.3. Environmental Characterization from Remote Sensing

The lechuguilla is generally harvested within a radius of 5 km around the inhabited localities. Therefore, this area was considered for the geo-climatic characterization of the three studied sites. Two environmental features (vegetation coverage and water availability) were described using Landsat 8 imagery. For this, two scenes (ID: 028042 for Cosme and Tuxtepec; ID: 028044 for Matehuala) were downloaded from EarthExplorer (https://earthexplorer.usgs.gov/) [accessed on 22 February 2021]. Normalized difference vegetation index (NDVI) was used as a proxy for vegetation biomass; usually this index takes 0 values for bare soils and 1 for highly dense rainforest. Normalized difference water index (NDWI) was used as a proxy for water-related factors such as water bodies and water stress [41]. Both indexes were obtained with R programing language (R Core Team, 2020) version 4.0.2, using raster package [42]. NDVI and NDWI values change depending on the vegetation type, season, and other landscape features; in this study, we used these values to compare the three sites on the same sampling date.

### 2.4. Phytochemical Extraction

The phytochemicals were obtained by ultrasound-assisted extraction (UAE) from 30 g of dried and milled residue, homogenized with 5 mL of ethanol/water (70/30, *v*/*v*) or methanol/water (60/40, *v*/*v*). The UAE was performed three consecutive times for 45 min, at 80.0 Hz and 40 °C; the solvent was collected and renewed between each incubation. The pooled supernatants (15 mL) were filtered at 0.22 µm (Whatman Uniflow Syringe Filters) and concentrated at 60 °C using a vacuum rotary evaporator (IKA, Willmington, NC, USA). Extracted phytochemicals were solubilized in approximatively 1 mL of distilled water, frozen at −80 °C, and freeze-dried for 24 h at −49 °C under vacuum. Finally, extraction yields were determined for each triplicate of ethanolic extract (EtOH) and methanolic extract (MetOH) and reported in relation to the fresh weight, according to the initial biomass moisture content.

### 2.5. Chemical Characterization

#### 2.5.1. Stock Solutions of Extracted Phytochemicals

For total polyphenol content (TPC) and total flavonoid content (TFC) determination, 10 mg of each triplicate of ethanolic and methanolic extract was recovered and solubilized in distilled water to reach a 10 mg/mL concentration. These stock solutions were used to prepare dilutions at 2 mg/mL, 1 mg/mL, and 0.5 mg/mL. For HPLC-UV-MS analyses, the ethanolic and methanolic extracts were dissolved at 1 mg/mL in methanol/water (50/50, *v*/*v*) HPLC-grade solvents and filtered through Whatman 0.45 µm nylon filters.

#### 2.5.2. Total Polyphenolic Content (TPC)

The TPC of the ethanolic and methanolic extracts was quantified according to Singleton and Rossi’s protocol [43], adapted to microplates. Briefly, 20 µL of extract dilutions, a negative control, and gallic acid solutions (from 0 to 500 mg/L) were placed in triplicate in a 96-well flat-bottom plate, 10 µL of Folin-Ciocalteu reagent (Sigma–Aldrich, St. Louis, MO, USA) was added, followed by 40 µL of Na_2_CO_3_ at 200 g/L and 130 µL of distilled water. After incubating for 30 min at 40 °C in the dark, the absorbance was read at 735 nm by the Epoch microplate reader (BioTek Instruments, Inc., Winooski, VT, USA). The phenol concentrations were obtained in milligram gallic acid equivalent (GAE) by reference to the standard curve (y=0.0085 x+0.0101, R² = 0.9965) and reported per gram of fresh weight (g FW), considering the moisture content and extraction yields.

#### 2.5.3. Total Flavonoid Content (TFC)

The TFC was determined in the extracts by the aluminum chloride method inspired by Lauranson-Broyer and Lebreton [44] and adapted to microplates. In brief, 20 µL of extract dilutions, negative control, and quercetin solutions (from 0 a 1400 mg/L), 7.5 µL of NaNO2 at 5%, 30 µL of 2.5% AlCl_3_ solution, 50 µL of NaOH at 1 M, and 50 µL of distilled water were deposited in this order into the 96-well flat-bottom plate, with 5 min homogenization between each addition. The absorbance was measured at 500 nm by the Epoch microplate reader (BioTek Instruments, Inc.). Flavonoid concentrations were estimated in milligrams quercetin equivalent by reference to the standard curve (y=0.0009 x+0.0451, R^2^ = 0.9928) and reported in relation to the fresh weight (mg QE/g FW).

#### 2.5.4. HPLC-UV-MS/MS

Apigenin, catechin, cyanidin, delphinidin, flavanone, hesperidin, isorhamnetin, kaempferol, naringenin, and quercetin in the ethanolic and methanolic extracts were quantified using flavonoid analytical standards (Sigma–Aldrich). All standards were prepared at 10–100 mg/mL in HPLC grade methanol/water (50/50, *v*/*v*), except for delphinidin and isorhamnetin, which were prepared at 5–50 mg/mL.

High-performance liquid chromatography (HPLC) was performed using Varian^®^ Pro Star HPLC equipment (Agilent Technology, Inc., Sta Clara, CA, USA), according to Méndez-Flores et al. [45]. In summary, 10 µL of the extracts and standards were injected through a Denali^®^ C18 column (250 mm × 4.6 mm size, and 5 µm particle size), maintained at 30 °C. A 0.2 mL/min flow was managed for 65 min, increasing the acetonitrile proportion linearly from 0 to 80% in the 0.2% formic acid aqueous mobile phase. Released compounds were detected under UV light at 280 nm through the photodiode array detector (PAD). The UV spectra were analyzed using the Agilent software Chromatography Workstation Star Toolbar (version 6.30).

At the same time, MS/MS analysis was performed to obtain the qualitative profiles of the flavonoids in the extracts. For that, the ionization was operated in negative polarity through an electrospray ionization (ESI-) system, and the mass spectra from 50–2000 *m*/*z* were recorded in full scan and MS/MS mode of the ion trap detector (Varian 500-MS IT Mass Spectrometer, Agilent). The MS/MS spectra were acquired with the Mass Spectrometry Workstation (version 6.9) software from Agilent for the Varian equipment.

### 2.6. Statistical Analysis

Analysis of variance (ANOVA) was used to assess the effect of the solvent and location on the extraction yield, TPC, and TFC. Quantitative HPLC-UV and Shapiro–Wilk test for normality and Bartlett test for homoscedasticity were performed to evaluate the two main statistical assumptions of ANOVA. Tukey HSD test was used to determine pairwise significant differences on levels of solvent and location. When normality was not present in the dependent variables, the Kruskal–Wallis test was used as a non-parametric alternative to ANOVA. All statistical tests were performed with an alpha of 0.05 in the R programing language (R Core Team, 2020) version 4.0.2.

## 3. Results

### 3.1. Geo-Climatic Conditions

**Figure 1:**
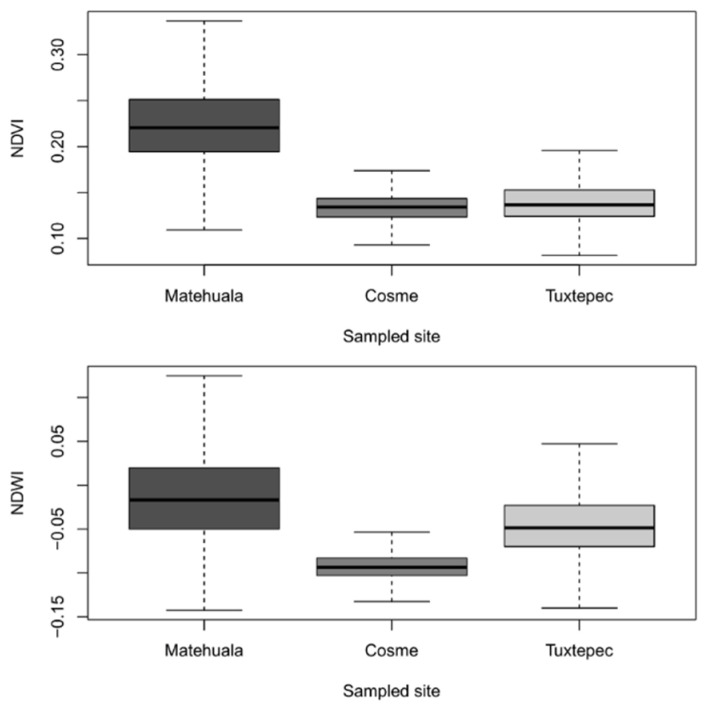
Statistics summary of normalized difference vegetation index (NDVI) and normalized difference water index (NDWI) values obtained by location from Landsat 8 imagery captured on 20 August 2018. Horizontal line = median, box = interquartile range (IQR), whiskers = 1.5 × IQR below first quartile or above third quartile.

**Figure 2:**
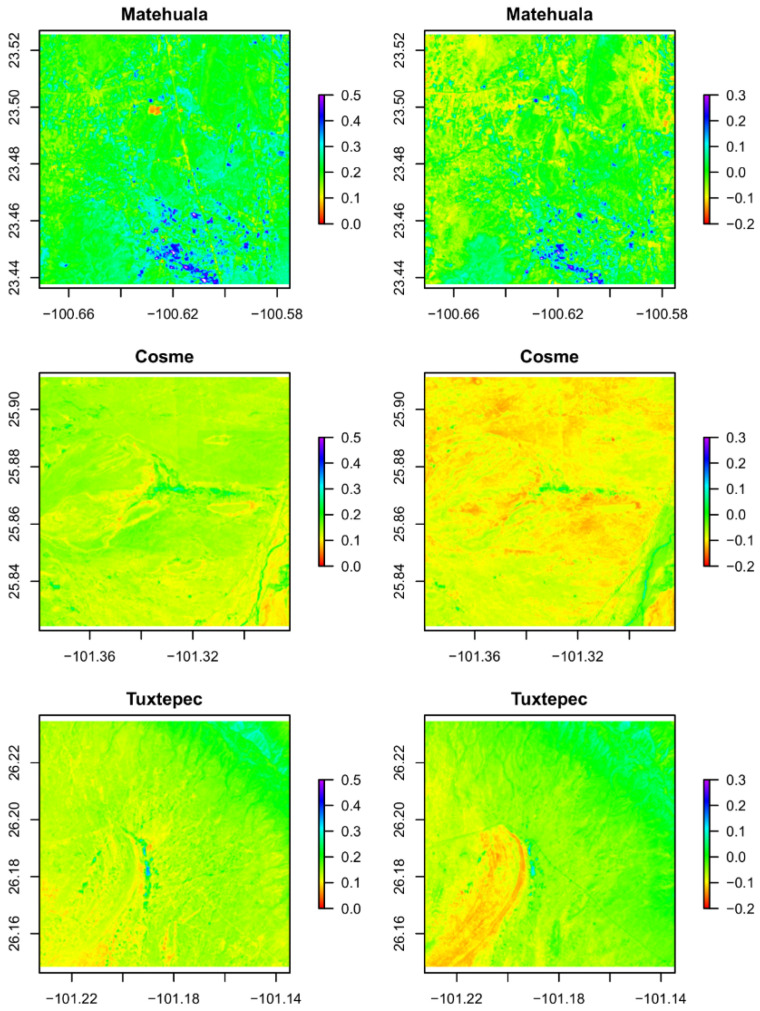
NDVI (left) and NDWI (right) values by location in the study area.

The three studied localities can be distinguished by their specific environmental features. NDVI values (Figure 1) suggest that vegetation cover is highest in Matehuala (San Luis Potosí), followed by Tuxtepec and Cosme (Coahuila), with average values of 0.22, 0.14, and 0.13, respectively. NDWI values (Figure 1 (bottom) and Figure 2 (right)) suggest that water stress is highest in Cosme, followed by Tuxtepec and Matehuala, with average values of −0.09, −0.04, and −0.01, respectively. In general, Cosme appeared as the most arid (less dense vegetation and dryer) location of this study.

### 3.2. Total Polyphenolic and Flavonoid Contents

**Figure 3:**
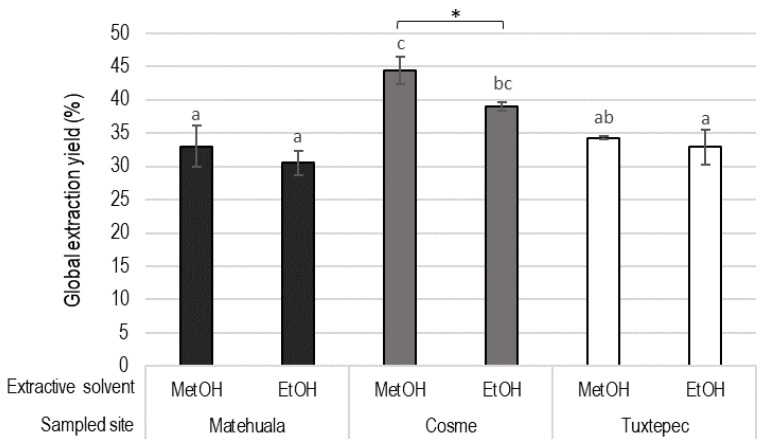
Extraction yields in percentage of dry biomass (%) obtained for methanolic (MetOH) and ethanolic (EtOH) extraction of *Agave lechuguilla* by-product from three different geographical sites. The letters show statistical significance between extracts (one-way ANOVA and Tukey post-hoc results, n = 3, *p*-value < 0.05), and * indicates solvent effect (two-way ANOVA, n = 3, *p*-value < 0.01).

The extraction yields of the polyphenolic compounds (Figure 3) were not significantly affected by the extractive solvent, resulting in 34.13 ± 4.11% for ethanolic extracts and 37.24 ± 5.73% for methanolic extracts. Comparing the three regions, independently from the solvent, Cosme presented a statistically higher extraction yield (41.71 ± 3.29%) than Tuxtepec and Matehuala, which had respective extraction yields of about 33.57 ± 1.80% and 31.78 ± 2.65%. Eventually, the highest extraction yield was obtained from the methanolic extract of the Cosme biomass (44.43 ± 2.06%).

**Figure 4:**
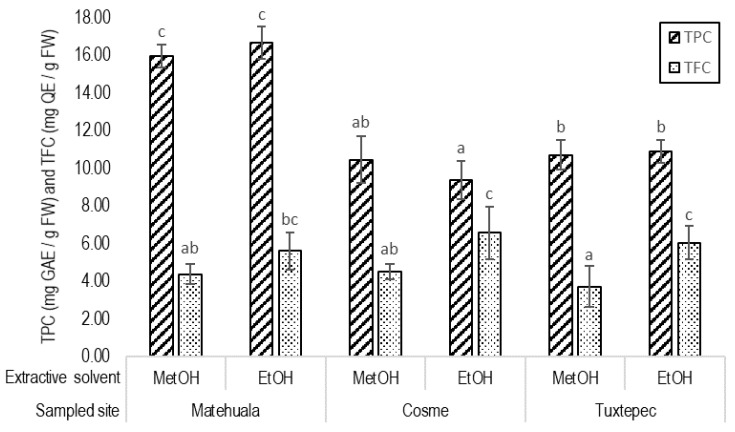
Total polyphenol content (TPC) expressed in milligrams gallic acid equivalent (GAE) and total flavonoid content (TFC) in quercetin equivalent (QE) per gram of fresh weight (FW) measured in methanolic (MetOH) and ethanolic (EtOH) extracts of *Agave lechuguilla* by-product from three different regions. Different letters indicate significant difference resulting from the one-way ANOVA and Tukey post-hoc test, respectively, performed on TPC and TFC (n = 12, *p*-value < 0.05).

As observed for extraction yields, the used solvent did not significantly impact the total polyphenolic content of the extracts. On the contrary, the total flavonoid contents were higher in ethanolic extracts than in methanolic extracts, independently from the studied site (Figure 4). According to the geo-climatic variables, the highest phenolic content was found for the Matehuala locality with TPC values reaching from 15.37 to 16.55 mg GAE/g FW in methanolic extracts and 15.83 to 17.55 mg GAE/g FW in ethanolic extracts. There was no difference between the TPC of the two sampled localities from the state of Coahuila (Cosme and Tuxtepec). The concentration of total polyphenols in Cosme methanolic and ethanolic extracts reached, respectively, 10.46 ± 1.23 mg GAE/g FW and 9.40 ± 1.23 mg GAE/g FW. The Tuxtepec extracts showed a TPC of about 10.72 + 0.79 mg GAE/g FW with methanol and 10.89 ± 0.61 mg GAE/g FW with ethanol (Figure 4). In contrast, there was no variation of the TFC due to the regional factor. The methanolic extracts showed a TFC from 3.74 ± 1.07 mg QE/g FW (Tuxtepec) to 4.53 ± 0.41 mg QE/g FW (Cosme), and the TFC of ethanolic fractions ranged from 5.62 ± 0.96 mg QE/g FW (Matehuala) to 6.58 ± 1.40 mg QE/g FW (Cosme) (Figure 4).

### 3.3. Flavonoid Profiles

The two-way ANOVA revealed that both location and extractive solvent impacted the concentrations of specific flavonoids in the extracts (Table 1). The organic solvent used to obtain flavonoid-enriched extracts significantly influenced the concentration of isorhamnetin and hesperidin, with 1216.65 ± 211.57 and 33.36 ±0.67 µg/g dry weight (DW), respectively, in methanolic extracts and 707.03 ± 74.66 and 4.25 ± 0.38 in ethanolic extracts (the regional factor explained deviations). In contrast, anthocyanins were only encountered in ethanolic fractions. Cyanidin ranged from 3.53 to 10.48 µg and delphinidin from 11.42 to 21.55 µg per gram of dry matter. The combination of all the quantified flavonoids showed a higher content for methanolic extracts (1655.86 µg/g DW). The ethanolic extracts exhibited higher diversity of the compounds (1028.12 µg/g DW).

**Figure 5:**
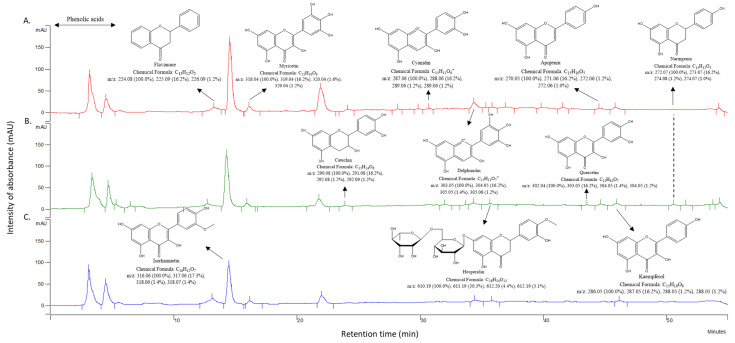
HPLC-UV spectra of ethanolic extracts of *Agave lechuguilla* by-product harvested from: (**A**) Matehuala, (**B**) Cosme, and (**C**) Tuxtepec, and lyophilized. Compound structure, name, and MS data were added using ChemDraw Professional (version 15.0) based on the result of Workstation Star Toolbar spectra analyses (Table 1 and Table 2) and analytical standards curve.

**Figure 6:**
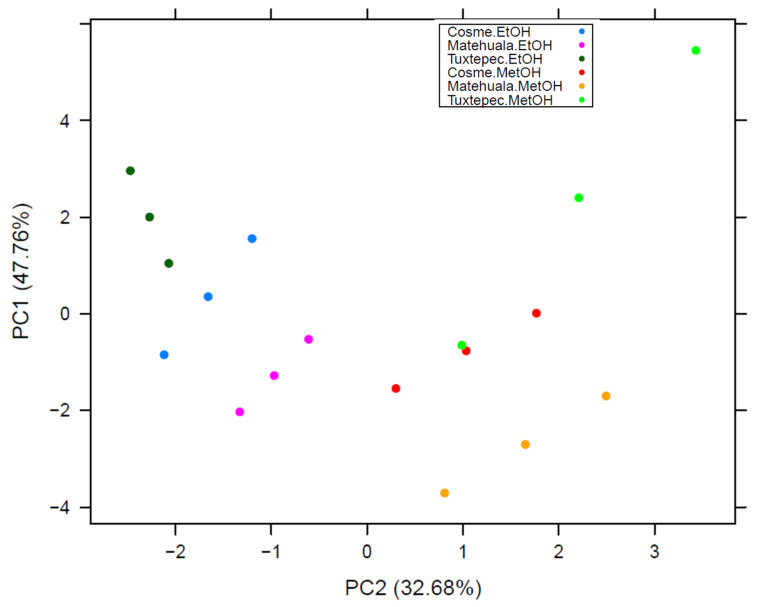
Plot of PCA analysis considering the flavonoids concentration in regard to extractive solvent and sampled site.

Regarding the geographical variable, flavonoid profiles were reflected in the HPLC-UV spectra of the ethanolic extracts. The presence of each quantified compound was corroborated through MS analysis (Figure 5). Flavonoid patterns in the ethanolic extracts distinguished the three localities, although the variation was nearly zero. The PCA analysis of flavonoid quantitative patterns according to regional and solvent factors did not clearly distinguish the three regional clusters due to the variation induced by the extractive solvent (Figure 6). Globally, the highest flavonoid concentration was obtained for Matehuala (1537.39 µg/g DW), followed by Tuxtepec (1332.35 µg/g DW) and Cosme (1156.21 µg/g DW). Matehuala was characterized by a greater abundance of flavanone and isorhamnetin, whereas Tuxtepec presented the highest concentrations of all other compounds, although only cyanidin and delphinidin contents were significant compared to Cosme (Table 1).

Individually, kaempferol averaged 12.98 ± 0.83 µg/g DW and was not affected by either the regional or the solvent factor. Apigenin and naringenin, which were among the lower concentrated compounds (8.26 ± 1.25 µg/g DW and 1.50 ± 0.23 µg/g DW, respectively) showed a similar trend, with only a slight difference (*p*-value < 0.05) in the methanolic extracts between Matehuala and Tuxtepec (Table 1). As mentioned above, hesperidin concentrations were higher in methanolic than in ethanolic extracts (*p*-value < 0.001) and were not different among the sampled localities. Similarly, isorhamnetin was found to be the methanolic extract with a significant abundance in Matehuala and Tuxtepec as compared to Cosme. In comparison, its presence in ethanolic extracts was the same among the sites (Table 1 and Figure 5). Both catechin and quercetin were higher in Tuxtepec methanolic extracts than in Matehuala extracts, although their concentrations were not significantly different regarding the region factor. The particular case of flavanone exhibited a regional variation according to the concentrations observed in methanolic extracts (*p*-value < 0.001), with higher content in Matehuala (520.13 ± 39.98 µg/g DW), and a lower range in Tuxtepec (214.10 ± 24.10 µg/g DW). The ethanolic extracts (261.66 ± 31.10 µg/g DW) did not exhibit a regional distinction (Table 1 and Figure 5). Finally, cyanidin and delphinidin detected exclusively in ethanolic extracts varied among regional factors. Their respective concentrations were twice as high in Tuxtepec than in Cosme (Table 1), whereas they could not be quantified in the Matehuala samples. However, they were found in the HPLC-UV spectra of the extracts obtained from the three sampled locations (Figure 5).

### 3.4. Conservation of Bioactive Flavonoids

**Figure 7:**
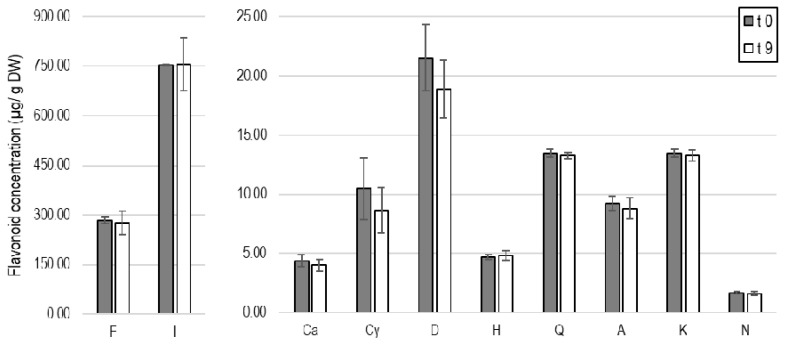
Concentrations (µg/g DW) of flavanone (F), isorhamnetin (I), catechin (Ca), cyanidin (Cy), delphinidin (D), hesperidin (H), quercetin (Q), apigenin (A), kaempferol (K), and naringenin (N), quantified by HPLC-UV (280 nm) analysis of ethanolic fractions extracted from Tuxtepec biomass after a few days of storage (t 0) and nine-month storage (t 9), preventing oxygen, moisture, and light exposure.

The Tuxtepec locality presented the highest diversity of quantified flavonoids. Therefore, the effect of storage conditions was evaluated based on the flavonoid content of the Tuxtepec biomass after nine months stored at room temperature, in the absence of light, oxygen, and moisture. Ethanolic extraction (ethanol/water, 70/30, *v*/*v*) of polyphenolic compounds was repeated. The extraction yield resulted in about 30.90 ± 1.86%, which was not statistically different (one-way ANOVA, n = 3, *p*-value > 0.05) from the yield previously obtained from residual biomass with no-storage time. Likewise, neither the TPC nor the TFC differed with storage time, with 10.40 ± 1.24 mg GAE/g FW and 5.93 ± 0.71 mg QE/g FW, respectively, found with the new extraction. Furthermore, the flavonoid profiles of the Tuxtepec agro-residue did not change significantly, although a decreasing tendency can be highlighted specifically for anthocyanin compounds (Figure 7). 

## 4. Discussion

### 4.1. Geo-Climatic Variation

Phenolic compounds, particularly the class of flavonoids, first known for their primary role as hormone regulators [46,47], are now better known for playing crucial ecophysiological roles in response to biotic and abiotic conditions [48,49]. *Agave lechuguilla* grows in xerophytic shrublands characterized by frequent drought events, nutrient-deficient soil, high salinity, extreme temperatures, and increased exposure to UV-radiations [50]. The wide distribution and density of *A. lechuguilla* in Mexico and the southern United States is due to its crassulacean acid metabolism (CAM) and its specialized metabolism [38,51,52]. Furthermore, along its distribution area, *A. lechuguilla* is faced with variable geo-climatic features. Regarding the total phenolic content, accumulation patterns varied according to the geographical origin, i.e., between Matehuala (San Luis Potosí) and the two sampled localities from Coahuila (Cosme and Tuxtepec), whereas the regional factor did not seem to affect the total flavonoid content (Figure 4). Likewise, the TPC and the TFC in *A. attenuata* leaf extracts show similar trends between the two different locations [53]. Despite the similar TFC, specific profiles in flavonoids quantified by HPLC-UV diverged among the geographical origin of the *A. lechuguilla* residue (Table 1). The environmental factors particularly influenced the accumulation of flavanone, methylated flavonoids (isorhamnetin), and anthocyanins (cyanidin and hesperidin) (Table 1). Additionally, the MS analysis, based on previous identification reported by Morreeuw et al. [39], revealed that glycoside derivatives content also differed between the three regions with 26 compounds in Tuxtepec, 24 in Cosme, and 20 in Matehuala (Table 2). Similarly, Almaraz-Abarca et al. [36] reported that glycoside derivatives of foliar phenolics varied between 12 and 20 by location, comparing height wild populations of *A. victoriae-reginae*. The difference observed in the flavonoid profiles revealed a specific biochemical adaptation strategy to the environmental conditions [40,50,54], which differ for the three sampled sites (Figure 1 and Figure 2 and Table 3).

Among the flavonoids synthesized by *A. lechuguilla*, the specific content in dihydroflavonoids and flavonols has already been suggested as being involved in drought adaptation, and it diverges from other *Agave* species [36]. In comparison, in *A. salmiana* grown under in vitro induced hydric stress, while the total polyphenol content is not affected, flavonol concentration decreases, and the specific content of kaempferol and quercetin glycosides are modulated [55]. The same authors also highlighted that, in vitro, specialized response to drought is different from in vivo due to the stress tolerance acquired by desert plants. Furthermore, the present results showed a lower concentration of catechin in Matehuala extracts (Table 1), which is the location with the highest averaged light exposure (Table 4). This observation is consistent with the fact that the UV-A and UV-B radiations enhance phenylalanine ammonia-lyase (PAL) activity, thus increasing the abundance of flavonoids, flavonols, anthocyanins, and condensed tannins; in the meantime, catechin, which is their precursor, decreases [56]. In this study, anthocyanidins and their glycosides, two quercetin glycosides, and two isorhamnetin glycosides were not found in the agro-residue from Matehuala (Table 3.). The aglycon forms of those compounds were more abundant in Tuxtepec, the sampled region with the lowest precipitation (Table 4) and a lower water availability (NDWI) compared to Matehuala (Figure 1 and Figure 2). These results are supported by previous knowledge about the activation of flavonoids and anthocyanins synthesis by water deprivation [40,56]. Furthermore, the higher anthocyanidin concentrations in Tuxtepec than in Cosme (Table 1) can be related to the differential UV-exposures between the two regions (Table 3). Anthocyanidins and derivatives have never been analyzed previously in *A. lechuguilla*, although, the current results coincide with geographical differentiation in global leaf color [57]. In contrast, a high temperature usually decreases anthocyanin content, which must be the main factor limiting anthocyanin accumulation in *A. lechuguilla* compared to non-desertic plants [56].

In addition, *A. lechuguilla* grows in a broad diversity of substrates, usually characterized by macronutrient deficiency and high concentrations of metallic ions, affecting the phytochemical composition [8]. The magnesium (Mg) deficiency symptom [54] was observed in the oldest leaves of organisms collected at the Cosme site and could be correlated with polyphenolic accumulation [56]. However, no specific accumulation pattern of the quantified flavonoids could be highlighted. To overcome the lack of nitrogen (N), some biomolecules can promote nitrogen-fixing bacterial expression [58]. This is the case in flavonoids such as naringenin, quercetin, and kaempferol (Table 1), as well as the rutin derivatives found in MS spectra of the three regions (Table 2), and the isoflavonoids, daidzein and genistein, which have also been found in *A. lechuguilla* specialized metabolic pathways [39]. It has been demonstrated that these flavonoids increased colonization and survival of the *A. lechuguilla* by acting as specific transmitters in symbiotic interactions with microorganisms, in particular, from the rhizosphere [59]. Likewise, naringenin content, which was stable among the regional factors (Table 1), has already been proven to have a positive effect on beneficial mycorrhizal fungi growth. In contrast, quercetin, also stable among samples (Table 1), inhibits harmful fungi [58]. On the other hand, micronutrients such as copper, zinc, iron, aluminum, and boron are also essential for *Agave lechuguilla’s* development. However, they are usually found at high even toxic levels, leading to growth inhibition [54,56]. As a response, *A. lechuguilla* presents the capacity to accumulate heavy metals using highly hydroxylated flavonoids such as isorhamnetin, flavanones, quercetin, and catechin (Table 1). These flavonoids are known for having high affinity with metallic ions, resulting in their chelation and neutralization [34]. Moreover, these flavonoids and their sulfated, methylated, and glycosylated derivatives that accumulate in *A. lechuguilla* [39] are considered osmotic metabolites when stored in the vacuole and cytoplasm or linked to the cell wall [40,56]; thus, they can provide better tolerance to drought and salt stress compared to other *Agave* species.

In addition to the abiotic conditions, *A. lechuguilla* also has to respond to biotic pressures. Allelopathic interactions are crucial in hyper-diversified ecosystems such as the xerophytic shrublands, where the lechuguilla grows [60,61]. Among the flavonoids found in the *A. lechuguilla* residue (Table 1 and Table 2) the flavone, catechin, and O-glycoside derivatives were previously identified as being involved in seed germination inhibition [62]. The higher concentration of catechin found in the Cosme and Tuxtepec sites (Table 1) can be related with the denser population of *A. lechuguilla* [8,54] and the lower global vegetation cover compared to Matehuala (Figure 1 and Figure 2). Additionally, associated species in the sampled sites differ between San Luis Potosí and Coahuila (Table 3). The allelopathic properties of the same flavonoids found in *A. lechuguilla* residue could also explain the environmental impact of their accumulation and why the areas where the lechuguilla residue is discarded does not show new plant growth for a while.

### 4.2. Agronomical and Biotechnological Implications

Despite the difference in the quantified flavonoids, explained by environmental features, the global accumulation pattern (TPC and TFC) suggests the potential of *A. lechuguilla* agro-industrial waste from the three productive regions as a source of natural compounds instead of being wasted. In addition, the flavonoids, mentioned above for their intrinsic biological activities involved in the physiological adaptation of *A. lechuguilla*, are also known for their wide range of bioactivities [24,25,28,29,32,34,35,63,64,65,66], which supports the current proposal to use it to obtain natural active ingredients.

The biorefinery model proposed by [2] separated saponins (26.0%) from other phytochemicals (10.4%). The extraction yields for phytochemicals (35.69 ± 5.09%) and the polyphenolic specific recovery range (12.28 ± 2.99%) obtained in this study agree with the phytochemical fraction previously estimated [2]. Besides, the polyphenolic concentrations obtained for *A. lechuguilla* agro-residue (Figure 4) reached higher values than those obtained for *A. attenuata*, with 1.05–3.93 mg GAE/g FW [64], and similar values to *A. americana*, with 2.37–18.72 mg GAE/g FW [53], and *A. ornithobroma*, with an average of 12.37 mg GAE/g FW [65]. Similarly, the flavonoid concentrations of *A. lechuguilla* (Figure 4) extracts were close to the TFC reported for *A. attenuata* and *A. americana* leaf extracts, with 0.43–3.05 mg CE/g FW [64] and 0.11–3.76 mg CE/g FW [53], respectively. 

Hence, as well as saponins, flavonoids are another high-value coproduct from *Agave lechuguilla*, cultivated primarily as fiber or bioenergy crops [38]. In addition, *A. lechuguilla* presents the advantage of not competing with food crops and products for agricultural land [2,52,66]. In this perspective, both economic and ecological approaches need to be enhanced for sustainable *A. lechuguilla* crops [67], finding a balance between productivity, fiber quality [5], and phytochemical content. In this regard, the changes in flavonoid accumulation, through geographical factors, provided valuable information for the establishment of crops. Although nitrogenous fertilizing promotes the growth of *A. lechuguilla* and phosphorus application enhances root development [54], they can also modulate the flavonoid content. Likewise, if initial watering can help crop establishment [68], it has also been suggested that improved breeding culture under daily watering can lose drought adaptation due to the change in the flavonoid pathway [40]. Actually, daily watering of *A. lechuguilla* crops induces a metabolic shift from CAM to the C3 photosynthetic pathway [3,51]. Therefore, changes in photosynthetic metabolism impact the production of the sugar subunits produced by *A. lechuguilla*; thus, glycoside flavonoid profiles are modulated by changing the main occurrence of arabinose, galactose, rhamnose, and xylose moiety [54]. This statement was evidenced by a higher amount of flavonoid with more than one glycoside moiety in the *A. lechuguilla* residue from the driest localities, Cosme and Tuxtepec, whereas quercetin-3-O-xylose was absent in the agro-residue from Matehuala (Table 2). In addition, the fluctuation of flavonoid profiles due to water availability directly impacts the biological properties of the derivate extracts.

To reach industrial applications, the flavonoid-enriched bioactive extracts have to be obtained using processes that meet always stricter local and international requirements about solvent traces in the final product and the limit of generated toxic residues [69]. In this respect, the results revealed that the extractive solvent impacted the flavonoid profiles rather than the geographical origin of the biomass (Figure 4 and Figure 6), which agrees with the same conclusions stated for *A. americana* [53]. The extraction yield was higher with methanol (Figure 1), whereas the maximum values of TPC and TFC were observed in ethanolic extracts (Figure 4). In comparison, the methanolic extract of *Agave* leaves exhibited the highest amount of TPC and TFC [64], although the impact of using ethanol compared to methanol to obtain an active fraction enriched in polyphenolic compounds was not considered. In *A. lechuguilla*, in contrast to TFC results, the total of quantified flavonoids was significantly higher in the methanolic extracts (1.66 ± 0.3 mg/g DW) than in the ethanolic extracts (1.03 ± 0.1 mg/g DW). The methanolic extracts revealed higher concentrations of isorhamnetin and hesperidin, compared to ethanol (Table 1), which is due to the differential polarity of the two hydroalcoholic mixes [36,64]. Ethanol and methanol present similar polarity and are effective for extracting flavonoids when they are in a mixture with water [53]. However, in this study, the ethanol/water mixture was 70/30 *(v*/*v*; EtOH), while the methanol/water was used at 60/40 (MetOH); therefore, their polarity differs. That is why the glycoside derivatives were found in higher amounts in the more polar fraction, i.e., MetOH. The glycosyl moiety prevent the chelation with the AlCl_3_ used for TFC determination [44,70]; thus, the concentration of glycoside derivatives in methanolic fraction could minimized the TFC results. In addition, the anthocyanins were only detected in the ethanolic fractions (Table 1). The concentration of anthocyanins probably increased the TFC of the ethanolic fractions due to their high metal-chelating property [71]. That is also suggested by the absence of significant difference in TFC according to solvent for the Matehuala samples (Figure 4), which do not present anthocyanins (Table 1). On the other hand, the contrast between TFC (Figure 4) and total quantified flavonoids (Table 1) could also be attributed to non-quantified flavonoids such as myricetin, identified in the ethanolic extract (Table 2). The results promote the use of high throughput profiling methods, e.g., HPLC-UV, rather than indirect determination methods, to quantify flavonoids. Furthermore, the use of ethanol as a non-toxic solvent to obtain flavonoids from *Agave* is supported by the present results and the previous report for *A. fourcroydes* where flavonoids and anthocyanins were also found preferentially in ethanolic extracts [72].

Finally, the agro-residue management as a source of flavonoids has been evaluated to guarantee the potential implementation on a larger scale while taking processing times into account. A greater diversity of flavonoids and higher specific concentrations (Table 2) were observed in the Tuxtepec agro-residue, particularly in the ethanolic fractions. Then, the Tuxtepec biomass was used to evaluate the reproducibility of extraction yields, TPC, TFC, and HPLC-UV-MS/MS profiles from t0 to t9 (months). Results evidenced that flavonoids proportion among phenolic content remains constant, and specific abundances were preserved after 9-month storage (Figure 7), suggesting the suitable conservation of flavonoids by controlling temperature, moisture, oxygen, and light exposure. Similarly, the ultrasound-assisted extraction performed in *A. fourcroydes* biomass stored for 6 months under the same conditions as in this study ranged similar concentrations of flavonoids and anthocyanins as the no storage time biomass [72]. Finally, the recommendation to store the biomass in the absence of light, moisture, and oxygen to ensure the adequate conservation of the bioactive flavonoids should be the starting point to promote bioprocess proposals.

## 5. Conclusions

The global accumulation patterns of phenolics and flavonoids, which remained stable over the studied geographical distribution of *Agave lechuguilla*, confirmed the potential use of the constantly produced agro-residue as a novel source of polyphenols-enriched extracts. However, variations in the specific abundance of the analyzed flavonoids was related to the environmental features, which differed among the three sampled locations. Further research must be carried out to precisely determine the effect of independent biotic and abiotic variables on the flavonoid accumulation patterns. Then, recommendations towards crop management to improve the balance between production and metabolite content is further advised.

This work also draws conclusions about the conservation of flavonoid profiles in conditioned agro-residues of *A. lechuguilla* and the constant free-radical scavenging capacity of the ethanolic extracts. These first insights about agro-residue processing provide useful information that will help in the development of bioprocesses for this raw material to improve the biorefinery of *A. lechuguilla*. In this regard, future analysis must be conducted to ensure the possible use of *A. lechuguilla* flavonoids as a functional ingredient in the cosmetic, nutraceutical, and pharma industry, and for application in emerging markets.

## Figures and Tables

**Table 1:** Flavonoid concentration (µg/g DW) measured by quantitative HPLC-UV using analytical standards (compounds) in ethanolic (EtOH) and methanolic (MetOH) extracts of *Agave lechuguilla* by-product from three different sampled sites (Matehuala, Tuxtepec and Cosme).

Peak N°	Compounds	Concentration (µg/g DW)					
				Site			
		Matehuala		Cosme		Tuxtepec	
				Extractive Solvent			
		MetOH	EtOH	MetOH	EtOH	MetOH	EtOH
1	Flavanone **	520.13 ± 39.98 ^c^	276.19 ± 16.35 ^ab^	359.86 ± 55.98 ^b^	224.78 ± 19.59 ^a^	214.10 ± 24.10 ^a^	284.01 ± 10.61 ^ab^
2	Isorhamnetin **	1416.70 ± 150.32 ^c^	753.97 ± 48.25 ^a^	981.55 ± 89.85 ^b^	614.32 ± 24.94 ^a^	1251.96 ± 58.24 ^c^	752.80 ± 2.91 ^a^
4	Catechin *	2.79 ± 0.26 ^a^	1.80 ± 0.07 ^a^	3.98 ± 1.30 ^ab^	4.97 ± 2.26 ^ab^	7.91 ± 3.62 ^b^	4.38 ± 0.50 ^ab^
5	Cyanidin **	0.00 ^a^	0.00 ^a^	0.00 ^a^	3.53 ± 0.22 ^b^	0.00 ^a^	10.48 ± 2.61 ^c^
6	Delphinidin **	0.00 ^a^	0.00 ^a^	0.00 ^a^	11.42 ± 0.32 ^b^	0.00 ^a^	21.55 ± 3.79 ^c^
7	Hesperidin **	32.90 ± 4.54 ^b^	4.00 ± 0.13 ^a^	32.96 ± 3.73 ^b^	4.05 ± 0.29 ^a^	34.23 ± 0.95 ^b^	4.69 ± 0.19 ^a^
8	Quercetin *	12.08 ± 0.69 ^a^	11.65 ± 0.66 ^a^	13.24 ± 0.80 ^ab^	13.73 ± 0.25 ^ab^	15.57 ± 2.47 ^b^	13.48 ± 0.32 ^ab^
9	Apigenin *	6.93 ± 0.29 ^a^	7.88 ± 0.33 ^ab^	7.95 ± 0.54 ^ab^	7.85 ± 0.84 ^ab^	9.70 ± 1.96 ^b^	9.25 ± 0.61 ^ab^
10	Kaempferol	12.29 ± 1.37 ^a^	12.78 ± 0.75 ^a^	12.82 ± 0.27 ^a^	12.77 ± 0.43 ^a^	13.71 ± 1.01 ^a^	13.48 ± 0.31 ^a^
11	Naringenin *	1.26 ± 0.08 ^a^	1.43 ± 0.07 ^ab^	1.45 ± 0.10 ^ab^	1.43 ± 0.15 ^ab^	1.76 ± 0.36 ^b^	1.68 ± 0.11 ^ab^
Total		2005.08	1069.70	1413.56	898.85	1548.94	1115.80

The same superscript letter within a line indicates no significant difference (two-way ANOVA, n = 3, *p*-value < 0.05 *, < 0.001 **.

**Table 2:** Qualitative profile of flavonoid compounds in guishe ethanolic extract. Experimental *m*/*z* and major fragments were obtained by HPLC-MS analysis in negative polarity ([M − H]^−^).

Compounds ^1^	*m*/*z* ([M − H]^−^ Fragments)	Matehuala	Cosme	Tuxtepec
Apigenin	268.9 (203.9)	+	+	+
Apigenin 7-O-glycoside	431.1 (268.9; 203.9)	+	+	+
Apigenin 7-O-rutinoside	578.5 (268.9)	+	+	+
Catechin	288.9	+	+	+
Cyanidin	287.1	−	+	+
Cyanidin 3-O-glycoside	478.4 (287.1)	−	+	+
Cyanidin O-diglycoside	609.2 (449.3; 287.1)	+	+	+
Delphinidin	319.1	−	+	+
Delphinidin 3-O-glycoside	465.4 (319.0)	−	+	+
Flavanone	223.1	+	+	+
Hesperidin	610.2	+	+	+
Isorhamnetin	316.1	+	+	+
Isorhamnetin-glycoside	478.4 (272.1)	+	+	+
Isorhamnetin 3-O-rutinoside	622.3 (146.1)	+	+	+
Isorhamnetin diglycoside 1	579.4 (316.1; 272.1)	−	+	+
Isorhamnetin diglycoside 2	609.2 (449.1; 272.1; 146.1)	+	+	+
Isorhamnetin triglycoside 1	756.5 (162.8; 146.1)	−	+	+
Isorhamnetin triglycoside 2	801.6 (316.1)	+	−	+
Kaempferol	285.1 (153.0; 107.0)	+	+	+
Kaempoferol 3-O-glycoside	431.1	+	+	+
Kaempferol 3-O-rutinoside	593.4 (285.1; 431.1)	+	+	+
Kaempoferol diglycoside	595.1 (285.1)	+	+	+
Kaempoferol triglycoside	772.5 (285.0)	+	+	+
Myricetin	317.0	+	+	+
Myricetin 3-O-glycoside	463.1 (317.0; 179.1)	+	-	+
Myricetin diglycoside	625.2 (317.0)	+	+	+
Naringenin	270.9	+	+	+
Naringenin O-rutinoside	579.5 (270.9)	+	+	+
Quercetin		+	+	+
Quercetin-3-O-xyloside	433.2 (300.9; 179.1)	+	+	+
Quercetin 3-O-glycoside 1	447.1 (301.0; 146.1)	+	+	+
Quercetin 3-O-glycoside 2	463.1 (301.0; 177.0)	−	+	+
Quercetin 3-O-rutinoside	609.2 (300.9; 463.1; 146.1)	+	+	+
Quercetin 3-O-diglycoside 1	595.1 (300.9; 447.1)	+	+	+
Quercetin 3-O-diglycoside 2	595.1 (300.9; 463.1)	−	+	+

^1^ Compounds identified by Morreeuw et al. (2021).

**Table 3:** *Agave lechuguilla* Torr. associated species in the harvesting area.

Matehuala (San Luis Potosi)		Cosme-Tuxtepec (Coahuila)	
Scientific Name	Family	Scientific Name	Family
Hechtia texensis S. Watson	Bromeliaceae	Larrea tridentata (DC.) Colville	Zygophyllaceae
Karwinskia humboldtiana (Schult.) Zucc.	Rhamnaceae	Fouquieria splendens Engelm.	Fouquieriaceae
Yucca carnerosana (Trel.) McKelvey	Asparagaceae	Euphorbia antisyphilitica Zucc.	Euphorbiaceae
Echinocactus platyacantus Link and Otto	Cactaceae	Hechtia texensis S. Watson	Bromeliaceae
Jatropha dioica Sessé ex Cerv.	Euphorbiaceae	Opuntia microdasys (Lehm.) Pfeiff.	Cactaceae
Leucophyllum laevigatum Standl.	Scrophulariaceae	Flourensia cernua DC.	Asteraceae
Viguieria stenoloba S.F. Blake	Asteraceae	Echinocereus stramineus (Engelm.) Rümpler	Cactaceae
Prosopis glandulosa (DC.) F.M. Knuth	Fabaceae	Cylindropuntia leptocaulis (DC.) F.M. Knuth	Cactaceae
Notholaena sinuata (Lag. ex Sw.) Kaulf.	Pteridaceae	Cylindropuntia kleiniae (DC.) F.M. Knuth	Cactaceae
Cylindropuntia kleiniae (DC.) F.M. Knuth	Cactaceae	Acacia berlandieri Benth.	Fabaceae
Larrea tridentata (DC.) Coville	Zygophyllaceae	Vachellia constricta (Benth.) Seigler and Ebinger	Fabaceae
Cylindropuntia imbricata (Haw.) F.M. Knuth	Cactaceae	Epithelantha micromeris (Engelm.) F.A.C. Weber ex Britton & Rose	Cactaceae
		Agave striata subsp. falcata (Engelm.) Gentry	Asparagaceae
		Echinocereus conglomeratus C.F. Först.	Cactaceae
		Jatropha dioica Sessé ex Cerv	Euphorbiaceae

**Table 4:** Average geo-climatic conditions in August for the three sampled sites according to the CONAGUA climatic database (https://smn.conagua.gob.mx/) (accessed on accessed on 22 February 2021).

Environmental Parameter	Matehuala	Cosme	Tuxtepec
Location	23°28′54.3″ N; 100°37′22.1″ W	25°52′03.6″ N; 101°19′51.1″ W	26°11′29.8″ N; 101°11′0.96″ W
Temperature	16.0–29.0 °C; Max 35 °C	18.8–30.6 °C; Max 40 °C	19.7–32.9 °C; Max, 35.9 °C
Photoperiod	13 h 12 min–12 h 36 min	13 h 18 min–12 h 39 min	13 h 18 min–12 h 39 min
Cloudy	36–42%	47–49% cloudy	47–49%
Precipitation	55.6 mm	44.8 mm	29.0 mm
Rainy days	4.3 days	2.8 days	3.4 days

## Data Availability

All data, belongs to this work, is given and presented herein the manuscript.
